# Temperature-Associated Prevalence and Multidrug Resistance of *bla*_NDM_-Positive *E. coli* in Livestock Farms in Xinjiang, China

**DOI:** 10.3390/ani16142113

**Published:** 2026-07-08

**Authors:** Shuqin Xu, Wansen Nie, Panpan Xia, Wanzhao Chen, Rui Tian, Mengqi Yang, Lining Xia

**Affiliations:** 1 College of Veterinary Medicine, Xinjiang Agricultural University, Urumqi 830052, China; 2 Xinjiang Key Laboratory of New Drug Research and Development for Herbivorous Animals (XJ-LNDRDHA), Urumqi 830052, China

**Keywords:** *bla*_NDM_-positive *E. coli*, temperature, antimicrobial resistance, resistance gene, Xinjiang

## Abstract

The dissemination of New Delhi metallo-β-lactamase (NDM)-producing *Escherichia coli* poses a serious threat to public health. Although increasing attention has been paid to the relationship between temperature and antimicrobial resistance (AMR) transmission, research in China’s Xinjiang region remains scarce. In this study, we conducted a cross-sectional survey of 1914 samples from intensive livestock farms across Xinjiang to characterize the detection rate and antimicrobial resistance profiles of *bla*_NDM_-positive *E. coli*. Using selective culture, PCR, and 16S rRNA sequencing, we identified 140 *bla*_NDM_-positive *E. coli* (7.3%), predominantly in northern Xinjiang. The highest detection rate of *bla*_NDM_-positive *E. coli* was found in Tacheng Prefecture (24.0%), followed by Changji Hui Autonomous Prefecture (4.3%) and Bortala Mongol Autonomous Prefecture (0.6%). No *bla*_NDM_-positive *E. coli* isolates were detected in Bayingolin Mongol Autonomous Prefecture, Kashgar Prefecture, or from pigeon samples. Binomial logistic regression analysis revealed a significant positive association between regional temperature and the detection rate of *bla*_NDM_-positive *E. coli*. Genotypic analysis identified 35 distinct resistance gene profiles, with predominant resistance genes including *bla*_TEM_ (87.9%), *ant(3″)-Ia* (97.1%), *qnrS* (92.9%), *tet*(A) (95.0%), *sul1* (70.0%), *sul3* (85.0%), and *floR* (97.1%); *mcr-1* and *mcr-8* were not detected. Antibiotic susceptibility tests showed that all isolates were resistant to imipenem and exhibited universal resistance to ampicillin, ceftiofur, tetracycline, florfenicol, and enrofloxacin, whereas the majority remained susceptible to amikacin (99.3%), tigecycline (94.3%), and polymyxin (77.1%). These findings indicate that livestock farms, particularly laying-hen systems in northern Xinjiang, may serve as reservoirs of multidrug-resistant *bla*_NDM_-positive *E. coli*, and that elevated regional temperature is an environmental factor associated with higher detection rates, underscoring a potential climate-associated risk for the spread of AMR.

## Simple Summary

Carbapenem antibiotics are critical for treating serious infections, and the production of New Delhi metallo-β-lactamase (NDM) can lead to bacterial resistance to these drugs. Although the use of carbapenem antibiotics is strictly prohibited in veterinary clinical practice, antibiotic-resistant bacteria carrying the NDM gene remain widespread. The role of temperature in the spread of such antibiotic-resistant bacteria is poorly understood. We investigated the occurrence of NDM-producing *Escherichia coli* in livestock and poultry farms across Xinjiang, China, a region with distinct climate zones. We found 140 (7.3%) *bla*_NDM_-positive *E. coli* from 1914 samples, mainly in northern Xinjiang. The detection rate was highest in Tacheng (24.0%). These *bla*_NDM_-positive *E. coli* isolates are characterized by multidrug resistance and the carriage of multiple resistance genes. Our findings reveal that farms in northern Xinjiang, especially laying-hen operations, may serve as reservoirs of *bla*_NDM_-positive *E. coli*. A significant positive association was observed between regional temperature and the detection rates of *bla*_NDM_-positive *E. coli*, with higher detection rates occurring in areas characterized by higher temperatures or greater temperature fluctuations. This pattern suggests that temperature is an environmental factor associated with the occurrence of *bla*_NDM_-positive *E. coli*; however, causal relationships remain to be established. These results underscore the need for incorporating climatic factors into surveillance programs to better understand and manage the risks posed by antimicrobial resistance-surveillance programs that consider climatic factors to better predict and control resistance risks.

## 1. Introduction

Antimicrobial resistance (AMR) has been listed by the World Health Organization as one of the major public health threats to global health security [1]. Carbapenem antibiotics are widely regarded as the “last line of defense” for the treatment of severe infections caused by Gram-negative bacteria, and the production of carbapenemases represents one of the principal mechanisms underlying carbapenem resistance [2]. New Delhi metallo-β-lactamase (NDM) can hydrolyze nearly all β-lactam antibiotics, including carbapenems [3]. Since its first report in 2008, NDM has spread rapidly on a global scale and has been identified in a broad range of Enterobacterales, thereby constituting a major resistance gene of significant clinical and public health concern [4].

Although carbapenem antibiotics are strictly prohibited in veterinary clinical practice, numerous surveillance studies have demonstrated that *bla*_NDM_ is not only frequently detected in clinical isolates but is also widely distributed in livestock and poultry farming, food products, and agricultural as well as urban environments, exhibiting pronounced cross-sectoral transmission at the human–animal–environment interface [5]. As the primary host of *bla*_NDM_ and a major reservoir of antimicrobial resistance genes (ARGs), *Escherichia coli* is not only a commensal bacterium in the intestines of humans and animals but also an important opportunistic pathogen, thereby serving as a representative and informative indicator organism for AMR surveillance and transmission dynamics [6]. Once *bla*_NDM_-positive *E. coli* are present in livestock and poultry farming, they can spread to humans through the food chain, direct contact, and environmental media, posing a potential public health risk [7].

Current research has largely focused on the effects of antimicrobial usage, livestock management, and hygiene measures on the emergence and dissemination of AMR [8]. In contrast, in recent years, temperature has received increasing attention as a potential environmental factor. Temperature can indirectly or directly regulate the abundance and distribution of ARGs by influencing bacterial growth rates, community structure, stress responses, and the frequency of horizontal gene transfer (HGT) [9,10]. A growing body of epidemiological and ecological studies has documented a positive correlation between rising environmental temperatures and both the abundance of ARGs and the detection rate of antibiotic-resistant bacteria across different regions and ecosystems [11]. Nevertheless, research on the temperature regulation of *bla*_NDM_ in livestock and poultry farming remains scarce.

Xinjiang, located in northwest China, is characterized by a typical continental arid climate [12]. The Tianshan Mountains traverse its territory, dividing it into the northern and southern regions with markedly different climates: the northern Xinjiang experiences pronounced temperature fluctuations, whereas the southern Xinjiang maintains relatively stable temperatures [13]. This distinctive geographical and climatic setting provides favorable conditions for studying the relationship between temperature and the spread of antibiotic-resistant bacteria. Current understanding of the detection rate of carbapenem-resistant bacteria, particularly *bla*_NDM_-positive *E. coli*, in livestock, poultry, and farming environments within Xinjiang remains limited. The relationship between this detection rate and regional temperature variations has not yet been fully elucidated.

Motivated by the above scientific questions and the distinct regional characteristics, this study conducted an epidemiological investigation of *bla*_NDM_-positive *E. coli* using samples collected from livestock, poultry, and associated farm environments across different climatic zones in Xinjiang. We characterized the antimicrobial resistance phenotypes and resistance gene profiles of the isolates, and integrated corresponding regional temperature data to evaluate the relationship between regional temperature and the detection rate of *bla*_NDM_-positive *E. coli*. From a One Health perspective, this study aims to elucidate the interaction between regional temperature and AMR at the animal–environment interface, thereby providing scientific evidence for carbapenem resistance risk assessment and the formulation of targeted intervention strategies in Xinjiang and comparable climatic regions.

## 2. Materials and Methods

### 2.1. Sample Collection

**Figure 1:**
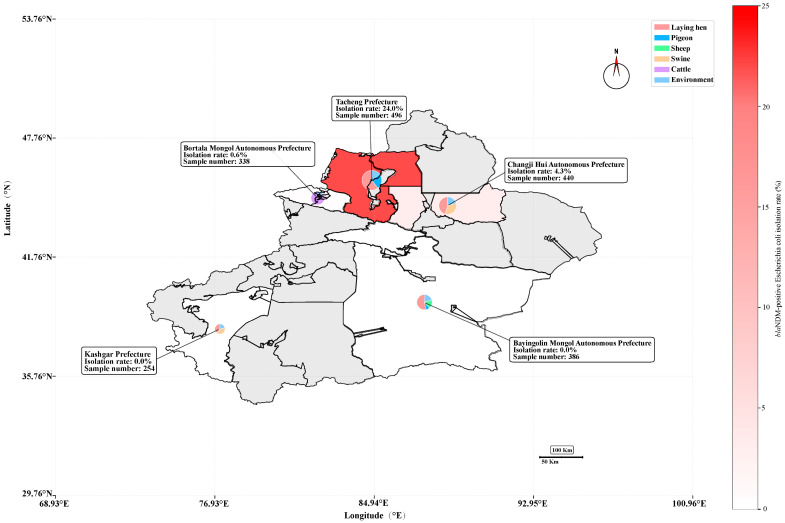
Detection rate of *bla*_NDM_-positive *E. coli*.

A total of 1914 samples, including 1579 fecal swabs and 335 environmental samples, were collected from livestock farms in Tacheng Prefecture, Changji Hui Autonomous Prefecture, Bortala Mongol Autonomous Prefecture, Bayingolin Mongol Autonomous Prefecture, and Kashgar Prefecture of Xinjiang, China during May to June 2024 (Figure 1). To ensure biological independence, each fecal sample was obtained from a different individual animal, with no animal sampled more than once. Specifically, 496 samples were obtained from Tacheng Prefecture, comprising fecal samples from laying hens (*n* = 300), laying-hen-farm environmental samples (*n* = 49), fecal samples from pigeons (*n* = 127), and pigeon-farm environmental samples (*n* = 20). A total of 440 samples were collected from Changji Hui Autonomous Prefecture, including fecal samples from laying hens (*n* = 200), laying-hen-farm environmental samples (*n* = 61), fecal samples from swine (*n* = 151), and swine environmental samples (*n* = 28). A total of 338 samples were collected from Bortala Mongol Autonomous Prefecture, consisting of cattle fecal samples (*n* = 294) and cattle-farm environmental samples (*n* = 44). In Bayingolin Mongol Autonomous Prefecture, 386 samples were obtained, including fecal samples from laying hens (*n* = 204), laying-hen-farm environmental samples (*n* = 57), fecal samples from pigeons (*n* = 33), pigeon-farm environmental samples (*n* = 12), fecal samples from sheep (*n* = 70), and sheep-farm environmental samples (*n* = 10). Finally, 254 samples were collected from Kashgar Prefecture, comprising fecal samples from laying hens (*n* = 100), laying-hen-farm environmental samples (*n* = 24), fecal samples from swine (*n* = 100), and swine-farm environmental samples (*n* = 30).

### 2.2. Isolation and Identification of bla_NDM_-Positive Isolates

All samples were inoculated into 1 mL of Brain Heart Infusion broth (Hope Bio, Qingdao, China) for bacterial enrichment and incubated at 37 °C for 24 h. Following enrichment, aliquots of the broth cultures were streaked onto MacConkey agar (Hope Bio, Qingdao, China) plates supplemented with imipenem (2 mg/L) using sterile inoculating loops. We selected 1~3 colonies with distinct morphologies and streaked them for purification until well-isolated carbapenem-resistant colonies were obtained. Putative carbapenem-resistant isolates were screened for the presence of the *bla*_NDM_ by polymerase chain reaction (PCR) amplification. Species identification of *bla*_NDM_-positive isolates was subsequently confirmed by 16S rRNA gene sequencing. A sample was defined as positive if at least one purified colony was confirmed as *bla*_NDM_-positive *E. coli*. To avoid duplicate analysis of clonal isolates from the same sample, only one *bla*_NDM_-positive *E. coli* isolate per positive sample was selected for subsequent characterization.

### 2.3. Statistical Analysis and Visualization

Daily average temperature data recorded over the 14 consecutive days prior to the sampling date were obtained from the national weather stations closest to the geographic coordinates of each farm (no on-farm microclimate measurements were available) [14]. The arithmetic mean of the collected daily average temperatures was used as the average temperature over the 14 days prior to sampling. Statistical analysis was performed in R (v4.3.0) using a binary logistic regression model (logit link function) to explore the relationship between the detection rate of *bla*_NDM_-positive *E. coli* and the regional average temperature over the 14 days prior to sampling. The model was fitted with a matrix of positive and negative counts as the response variable; results are reported as the regression coefficient with its 95% confidence interval (significance threshold: *p* < 0.05). The corresponding figure—generated with ggplot2—plots the observed detection rates (point size scaled by sample size) against the regional average temperature over the 14 days prior to sampling, and overlays the model-predicted probability curve with its 95% confidence band.

### 2.4. Antimicrobial Susceptibility Testing

Antimicrobial susceptibility testing of *bla*_NDM_-positive *E. coli* isolates was performed using the agar dilution method. A total of ten antimicrobial agents were evaluated, including imipenem (IPM), ampicillin (AMP), ceftiofur (CFF), polymyxin (PE), amikacin (AMK), gentamicin (GEN), enrofloxacin (ENR), tetracycline (TET), tigecycline (TIG), and florfenicol (FFC). Susceptibility patterns were interpreted according to the Clinical and Laboratory Standards Institute (CLSI) (https://clsi.org) breakpoint criteria and the guidelines of the European Committee on Antimicrobial Susceptibility Testing (EUCAST) (https://www.eucast.org/) for Enterobacteriaceae (Supplementary Table S1). Each test employed *E. coli* ATCC 25922 as the reference strain and quality control standard.

### 2.5. Identification of ARGs

ARGs representing eight antimicrobial classes were detected by PCR. These included β-lactam resistance genes (*bla*_TEM_, *bla*_CTX-M_, *bla*_SHV_), peptide resistance genes (*mcr-1*, *mcr-8*), aminoglycoside resistance genes (*ant(3″)-Ia*, *aac(6′)-Ib*), quinolone resistance genes (*oqxA*, *oqxB*, *qnrS*), tetracycline resistance genes (*tet*(A), *tet*(M)), sulfonamide resistance genes (*sul1*, *sul2*, *sul3*), and amphenicol resistance gene (*floR*). The primer sequences are provided in Supplementary Table S2.

## 3. Results

### 3.1. Isolation and Identification of bla_NDM_-Positive Isolates

A total of 140 *bla*_NDM_-positive *E. coli* isolates were recovered from 1914 samples (Figure 1). Of these, 119 (24%, 119/496) originated from the Tacheng Prefecture, including 118 (39.3%, 118/300) from laying-hen fecal samples and 1 (2%, 1/49) from the laying-hen-farm environmental samples. From Changji Hui Autonomous Prefecture, 19 isolates (4.3%, 19/440) were obtained: 13 (6.5%, 13/200) from laying-hen fecal samples, 5 (3.3%, 5/151) from swine fecal samples, and 1 (3.6%, 1/28) from the swine-farm environmental samples. Two isolates (0.6%, 2/338) were recovered from Bortala Mongol Autonomous Prefecture, both (0.7%, 2/294) from cattle fecal samples. No *bla*_NDM_-positive *E. coli* isolates were detected in any pigeon samples. Furthermore, *bla*_NDM_-positive *E. coli* was not isolated from any of the samples collected from laying hens, sheep, pigeons, swine, or associated environmental sources in Bayingolin Mongol Autonomous Prefecture or Kashgar Prefecture.

**Figure 2:**
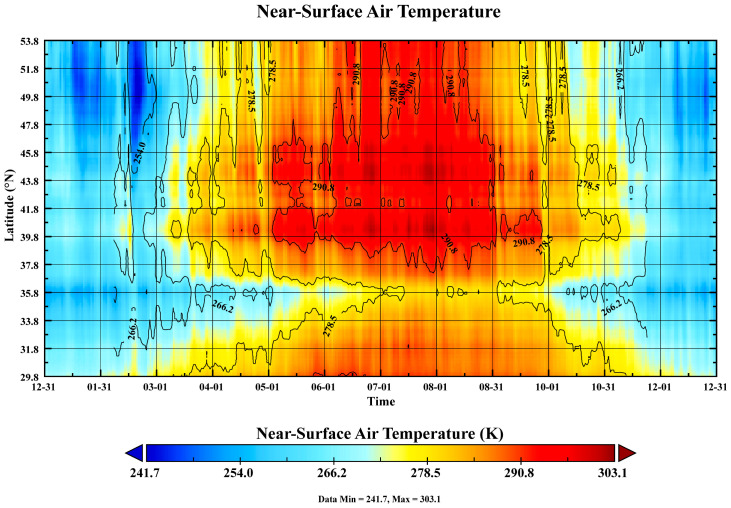
Spatio-temporal distribution of near-surface air temperature across mid-latitudes (29° N–54° N). Based on the one-year observation data, a spatio-temporal distribution heat map of near-surface air temperature is plotted. The color gradient from blue to red represents the variation in temperature from 241.7 K (minimum) to 303.1 K (maximum). The horizontal axis represents time (from the most recent December 31 backward for one year), and the vertical axis represents latitude (20° N to 53° N).

**Figure 3:**
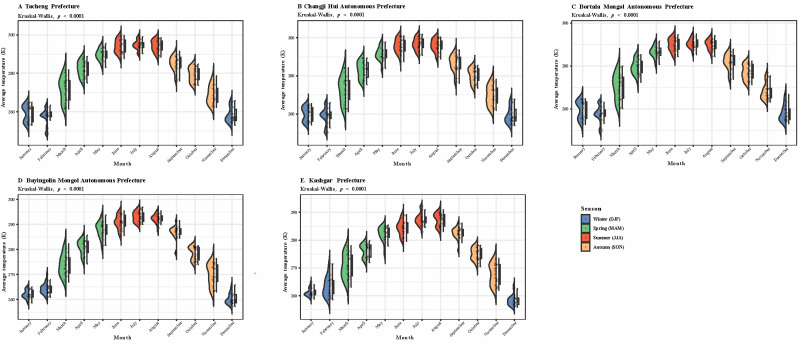
The average monthly temperatures of five regions. (**A**). Tacheng Prefecture. (**B**). Changji Hui Autonomous Prefecture. (**C**). Bortala Mongol Autonomous Prefecture. (**D**). Bayingolin Mongol Autonomous Prefecture. (**E**). Kashgar Prefecture.

**Figure 4:**
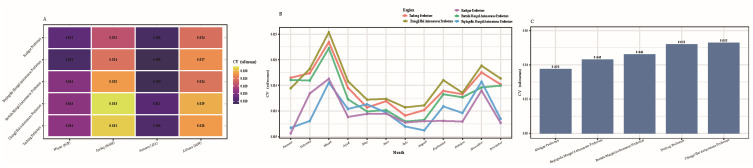
Temperature coefficient of variation. (**A**). Temperature Variability Coefficient by region and quarter. (**B**). Monthly temperature variation coefficient by region. (**C**). Overall temperature variability coefficient of five regions.

The detection rate of *bla*_NDM_-positive *E. coli* may be influenced by multiple factors, such as animal species, antimicrobial usage, farm management practices, and local environmental conditions. Xinjiang is characterized by a typical continental arid climate with pronounced seasonality. The Tianshan Mountains traverse central Xinjiang, dividing the region into northern and southern zones with distinct climatic patterns, particularly in temperature distribution. In northern Xinjiang, Tacheng Prefecture (46.75° N), Changji Hui Autonomous Prefecture (44° N), and Bortala Mongol Autonomous Prefecture (45° N) exhibit marked seasonal temperature fluctuations (Figure 2). In Tacheng Prefecture, the mean summer and winter temperatures are approximately 296.75 K and 257.35 K (Figure 3A), respectively, corresponding to a mean annual temperature of ~279.15 K and an annual temperature range of 39.38 K. In Changji Hui Autonomous Prefecture, the mean summer temperature is ~296.85 K and the mean winter temperature is ~259.15 K (Figure 3B), yielding a mean annual temperature of ~279.45 K and an annual range of 37.70 K. In Bortala Mongol Autonomous Prefecture, the mean summer and winter temperatures are ~293.35 K and ~262.95 K (Figure 3C), with a mean annual temperature of ~282.05 K and an annual temperature range of 30.40 K. By contrast, southern Xinjiang, represented by Bayingolin Mongol Autonomous Prefecture (42° N) and Kashgar Prefecture (39.5° N), shows comparatively smaller temperature variability. In Bayingolin Mongol Autonomous Prefecture, the mean summer temperature is ~293.05 K and the mean winter temperature is ~262.05 K (Figure 3D), with a mean annual temperature of ~278.43 K and an annual range of 32.78 K. In Kashgar Prefecture, the mean summer and winter temperatures are ~287.55 K and ~260.85 K (Figure 3E), with a mean annual temperature of ~274.73 K and an annual range of 29.13 K. A comprehensive temperature comparison across the five regions is provided in Supplementary Figure S1. Notably, the coefficient of variation in temperature declines progressively from Tacheng Prefecture to Kashgar Prefecture (Figure 4), indicating that temperature fluctuations become increasingly stable along this geographic gradient. Consistent with this trend, the detection rate of *bla*_NDM_-positive *E. coli* was substantially higher in regions with greater temperature variability and more pronounced seasonal extremes than in regions with comparatively stable temperatures. Together, these observations suggest that temperature variability may play a role in the abundance and dissemination of ARGs.

**Figure 5:**
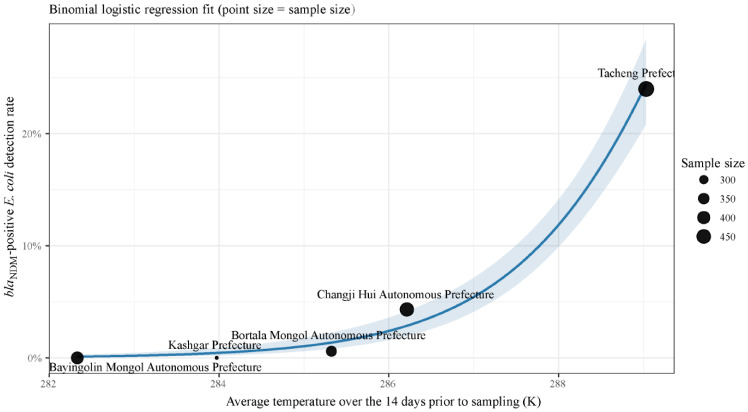
Binary logistic regression fitting.

A binomial logistic regression model was applied to evaluate the effect of regional temperature on the detection rate of *bla*_NDM_-positive *E. coli*. Statistically, the univariable logistic regression revealed a significant positive correlation between the 14-day mean temperature and the detection rate of *bla*_NDM_-positive *E. coli*. The regression coefficient for temperature was β = 0.8502 (SE = 0.0757, *p* < 0.001, 95% CI: 0.7108–1.0093). This yields an estimated odds ratio (OR) of 2.34 (95% CI: 2.04–2.74) per 1 K increase in ambient temperature. To quantify the temperature-dependent escalation of risk, we derived exact threshold temperatures using the inverse calculation of the fitted binomial logistic regression equation (ln(p/(1 − p)) = −246.8638 + 0.8502 × T). Based on this model, the temperature corresponding to a predicted low-level detection probability of 0.1% was 282.23 K, accounting for the near-zero detection rates observed at the colder sites (Figure 5). Furthermore, the inflection point where the slope steepened dramatically, corresponding to a predicted detection probability of 5%, was calculated to be 286.89 K. Beyond this threshold, the non-linear escalation in the risk of *bla*_NDM_-positive *E. coli* detection accelerated exponentially. This trend is corroborated by actual data. For instance, no *bla*_NDM_-positive *E. coli* were detected in regions with lower mean temperatures, such as Bayingolin Mongol Autonomous Prefecture (282.33 K, *n* = 386) and Kashgar Prefecture (283.97 K, *n* = 254). Conversely, the highest detection rate of 24.0% (119/496) was observed in Tacheng Prefecture, which recorded the highest 14-day mean temperature (289.03 K) among the surveyed areas. Collectively, these findings indicate a significant positive association between regional temperature and the detection rate of *bla*_NDM_-positive *E. coli*.

### 3.2. Antimicrobial Susceptibility Profiles

**Figure 6:**
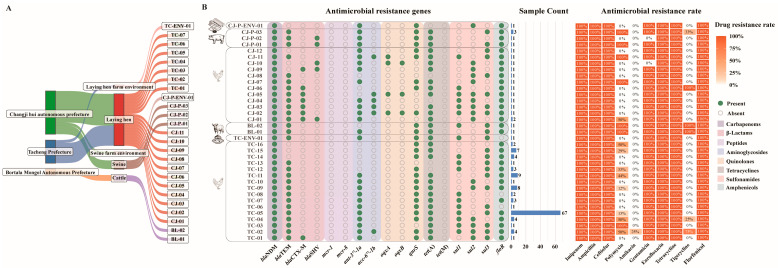
Antimicrobial resistance profile of *bla*_NDM_-positive *E. coli*. (**A**). Sources of *bla*_NDM_-positive *E. coli*. (**B**). Detection of ARGs and antimicrobial ausceptibility testing results for *bla*_NDM_-positive *E. coli*.

Among the 140 *bla*_NDM_-positive *E. coli* isolates, a total of 35 antibiotic resistance gene (ARG) subtypes were detected (Figure 6; raw data for ARG detection and antimicrobial susceptibility testing are provided in Supplementary Table S3). The detailed characteristics of representative isolate groups are summarized below. Sixty-seven *bla*_NDM_-positive *E. coli* isolates were obtained from laying hens in Tacheng Prefecture (TC-05 group). All 67 isolates carried *bla*_TEM_, *ant(3″)-Ia*, *qnrS*, *tet*(A), *tet*(M), *sul1*, *sul2*, and *floR*, and exhibited resistance to ampicillin, ceftiofur, gentamicin, enrofloxacin, tetracycline, and florfenicol; the resistance rate to polymyxin was 13%, while all isolates remained susceptible to amikacin and tigecycline. Nine additional isolates from laying hens in Tacheng Prefecture (TC-11 group) carried *bla*_TEM_, *ant(3″)-Ia*, *qnrS*, *tet*(A), *tet*(M), *sul2*, and *floR;* these isolates displayed a similar resistance profile but with a higher polymyxin resistance rate of 44%, and remained susceptible to amikacin and tigecycline. Two *bla*_NDM_-positive *E. coli* isolates were recovered from cattle in Bortala Mongol Autonomous Prefecture. Both were resistant to ampicillin, ceftiofur, polymyxin, gentamicin, enrofloxacin, tetracycline, tigecycline, and florfenicol, but susceptible to amikacin. The BL-01 isolate carried *bla*_TEM_, *ant(3″)-Ia*, *qnrS*, *tet*(A), *tet*(M), *sul3*, and *floR*, whereas the BL-02 isolate carried *bla*_TEM_, *qnrS*, *tet*(A), *tet*(M), *sul1*, *sul3*, and *floR*. One *bla*_NDM_-positive *E. coli* isolate from a laying hen in Changji Hui Autonomous Prefecture (CJ-02 group) carried *bla*_TEM_, *bla*_CTX-M_, *ant(3″)-Ia*, *aac(6′)-Ib*, *qnrS*, *tet*(A), *tet*(M), *sul1*, *sul2*, *sul3*, and *floR*. This isolate was resistant to ampicillin, ceftiofur, gentamicin, enrofloxacin, tetracycline, and florfenicol, but susceptible to polymyxin, amikacin, and tigecycline. Three *bla*_NDM_-positive *E. coli* isolates from swine in Changji Prefecture (CJ-P-03 group) uniformly carried *bla*_TEM_, *ant(3″)-Ia*, *qnrS*, *tet*(A), *sul3*, and *floR*, and displayed resistance to the same set of antibiotics while remaining susceptible to polymyxin, amikacin, and tigecycline.

Overall, the 140 *bla*_NDM_-positive *E. coli* isolates primarily carried *bla*_TEM_ (87.9%), *ant(3″)-Ia* (97.1%), *qnrS* (92.9%), *tet*(A) (95%), *sul1* (70%), *sul3* (85%), and *floR* (97.1%); *mcr-1* and *mcr-8* were not detected. Antimicrobial susceptibility testing revealed universal resistance to imipenem (100%), as well as complete resistance to ampicillin, ceftiofur, tetracycline, florfenicol, and enrofloxacin; gentamicin resistance reached 98.6%. In contrast, based on the antimicrobial susceptibility testing results, amikacin, tigecycline, and polymyxin retained comparatively high in vitro activity, with susceptibility rates of 99.3%, 94.3%, and 77.1%, respectively, suggesting their potential clinical utility in treating infections caused by *bla*_NDM_-positive isolates. Collectively, these findings demonstrate that *bla*_NDM_-positive *E. coli* harbor diverse ARGs and display multidrug-resistant phenotypes, underscoring the substantial burden of AMR in farm settings.

## 4. Discussion

The rapid dissemination of carbapenem-resistant Enterobacterales (CRE) poses a significant threat to global public health, with *bla*_NDM_ representing one of the most prevalent and clinically significant carbapenemase genes worldwide. In this study, we characterized the epidemiological distribution, antimicrobial resistance phenotypes, and genetic profiles of *bla*_NDM_-positive *E. coli* isolated from livestock, poultry, and their associated environments across multiple regions of Xinjiang. Additionally, we conducted an exploratory assessment of the relationship between regional temperature and the transmission dynamics of *bla*_NDM_-positive *E. coli*. These findings provide critical evidence to support risk assessment and the development of targeted strategies for the surveillance, prevention, and control of antibiotic-resistant bacteria at the regional level.

A total of 140 *bla*_NDM_-positive *E. coli* isolates were recovered from 1914 samples, confirming their presence of these isolates in livestock, poultry, and farm-associated environments in Xinjiang and highlighting the potential risk of dissemination within agroecosystems. Host distribution analysis revealed that layer hens constituted the dominant reservoir of *bla*_NDM_-positive *E. coli* in this region (95.7%, 134/140), with a significantly higher detection rate than that observed in other livestock species, including swine and cattle. This pattern is consistent with the findings of Fu et al. [15], who reported substantially higher detection rates of NDM-CRE in poultry than in swine, suggesting that *bla*_NDM_ transmission in the study area is likely concentrated within intensive poultry production systems. Consistent with previous reports by Tang M et al. [16] and Guan Y et al. [17], *bla*_NDM_-positive *E. coli* were also detected in poultry and swine farm environments. Environmental matrices such as manure, litter, wastewater, and equipment surfaces can serve as important reservoirs and transmission interfaces, promoting bacterial survival and the horizontal transfer of mobile genetic elements among different hosts. These findings underscore the role of the farm environment as a critical ecological niche for the maintenance and dissemination of AMR, rather than merely a passive recipient of antibiotic-resistant bacteria. Integration of our data with previously reported results reveals that the detection rate of NDM-CRE exhibits a marked increasing trend along the food production chain, rising from 4.70% at the farm level to 7.60% at slaughterhouses and 65.56% at retail [15]. This pattern suggests that AMR prevalence may progressively amplify at successive stages of the supply chain, ultimately facilitating transmission to consumers. This cumulative amplification markedly elevates the risk of human exposure and represents a substantial foodborne public health concern. Notably, all pigeon samples examined in this study tested negative for *bla*_NDM_-positive *E. coli*. This finding contrasts with reports from Jiangsu Province, China [18], and Jeddah, Saudi Arabia [19], where *bla*_NDM_-positive isolates were identified in pigeons. Such discrepancies suggest that the distribution of ARGs may be influenced by regional factors, including farming practices, antimicrobial usage, ecological conditions, and local management systems. In the present study, pigeons were raised under small-scale, free-range conditions, which may involve lower antimicrobial selection pressure and reduced opportunities for bacterial transmission. This observation highlights the importance of incorporating host species, production models, and regional ecological contexts into surveillance and intervention strategies, rather than directly extrapolating findings or control measures from other regions.

This study demonstrates that the overall detection rate of *bla*_NDM_-positive *E. coli* among farmed animals in Xinjiang is 7.31%. Given that carbapenems are regarded as the “last line of defense” for the treatment of multidrug-resistant (MDR) bacterial infections in clinical settings, this finding indicates a concerning prevalence of CRE contamination within the regional farming environment. The distribution of *bla*_NDM_-positive *E. coli* exhibited pronounced geographical heterogeneity: detection rates were significantly higher in regions north of the Tianshan Mountains than in those to the south, and no *bla*_NDM_-positive *E. coli* were identified in Bayingolin Mongol Autonomous Prefecture or Kashgar Prefecture in southern Xinjiang. This distribution pattern suggests that northern Xinjiang may represent a key region for the emergence and dissemination of *bla*_NDM_. Although factors such as clinical antimicrobial usage, biosecurity management levels, and production systems may contribute to regional differences in detection rates, the marked climatic disparities between northern and southern Xinjiang offer a novel environmental perspective from which to interpret this phenomenon. The Tianshan Mountains serve as a natural geographical barrier, creating distinct climatic conditions between the two regions. Our results indicate that regional temperature and its variability are strongly associated with the detection rate of *bla*_NDM_-positive *E. coli*. Specifically, northern Xinjiang is characterized by greater annual temperature fluctuations and a higher temperature coefficient of variation, and significantly higher detection rates of *bla*_NDM_-positive *E. coli*. In contrast, southern Xinjiang exhibits relatively stable temperature patterns and correspondingly low detection rates. These findings are consistent with global observations linking regional temperature to AMR. Supporting this association, Collignon et al. [20] demonstrated a significant positive correlation between ambient temperature and carbapenem resistance across 103 countries. MacFadden et al. [21] further quantified this relationship in the United States, showing that every 10 °C increase in minimum environmental temperature was associated with an approximate 4.2% increase in the resistance rate of *E. coli*. Similarly, McGough et al. [22] reported that, even after controlling for major confounders such as antibiotic consumption and population density, environmental temperature remained strongly associated with the temporal dynamics of AMR across Europe. This observed association underscores the importance of incorporating climatic and environmental variables into risk assessments and surveillance strategies for carbapenem resistance in agricultural settings.

From a One Health perspective, rising environmental temperatures linked to global climate change are accompanied by changes in agricultural ecosystems [23], animal health and welfare, human health, and the surrounding environment [24]. Magnano San Lio et al. [25] systematically investigated the relationship between rising environmental temperatures and the dissemination of AMR. Existing studies have also reported that elevated temperatures are associated with greater enrichment and persistence of ARGs in wastewater, soil, and aquaculture systems [26,27,28]. Consistent with these observations, this study found that the high detection rate of *bla*_NDM_-positive *E. coli* in northern Xinjiang is associated with regional temperature patterns, suggesting that the occurrence of these ARGs is linked to temperature as an environmental factor. Temperature, along with antimicrobial usage, farming practices, and ecological conditions, is one of multiple factors associated with microbial survival, community structure, and HGT, thereby facilitating the environmental reservoir and spread of clinically important ARGs such as *bla*_NDM_-positive *E. coli*. These findings underscore the necessity of integrating climatic variables into One Health–oriented surveillance and mitigation strategies for AMR.

All *bla*_NDM_-positive *E. coli* isolates identified in this study exhibited MDR phenotypes and commonly carried multiple ARGs, underscoring the complexity and severity of AMR within livestock farming. Antimicrobial susceptibility testing revealed that, in addition to resistance to imipenem, a representative carbapenem, these isolates displayed universal resistance (100%) to ampicillin, ceftiofur, tetracycline, florfenicol, and enrofloxacin, along with an extremely high resistance rate to gentamicin (98.6%). These findings indicate that many conventional broad-spectrum antimicrobials have largely lost their therapeutic effectiveness in this farming context. Notably, consistent with previous reports by Chakraborty T et al. [29] and Ma JX et al. [30], the present study found that a subset of *bla*_NDM_-positive *E. coli* remained highly susceptible to tigecycline, polymyxins, and amikacin according to the antimicrobial susceptibility testing results. This observation suggests that these drugs may serve as candidate agents for further clinical evaluation in treating infections caused by *bla*_NDM_-positive *E. coli*. Furthermore, resistance gene profiling revealed that genes such as *bla*_TEM_, *ant(3″)-Ia*, *qnrS*, *tet*(A), and *floR* were detected in more than 87% of the *bla*_NDM_-positive *E. coli* isolates. This high level of co-occurrence is consistent with findings reported by Wang MG et al. [31] and highlights the frequent linkage of *bla*_NDM_ with multiple ARGs. This further indicates that the *bla*_NDM_ gene is often co-present with multiple ARGs. These associated resistance genes are often co-located on the same plasmid or other mobile genetic elements, thereby facilitating their synergistic dissemination under the combined antimicrobial selective pressures. Collectively, these results emphasize the elevated risk of rapid spread and persistence of complex MDR genotypes in agricultural environments.

## 5. Conclusions

This study indicates that livestock and poultry production systems in northern Xinjiang, particularly laying-hen farms and their surrounding environments, may act as reservoirs of *bla*_NDM_-positive *E. coli*, thereby posing a potential threat to public health. Exploratory analysis further revealed a significant positive association between regional temperature and the detection rate of *bla*_NDM_-positive *E. coli* across northern and southern Xinjiang. The *bla*_NDM_-positive *E. coli* isolates characterized in this study exhibited multidrug resistance and harbored multiple ARGs, underscoring their capacity for persistence and transmission. Collectively, these findings highlight the urgent need to strengthen standardized management practices in livestock and poultry farming through a One Health perspective, in order to mitigate the risk of AMR dissemination. Furthermore, a long-term, systematic AMR surveillance framework should be established that incorporates climatic factors—particularly temperature variability—into risk assessment models, thereby enhancing the prediction, early warning, and control of AMR trends.

## 6. Research Limitations

First, owing to its cross-sectional design and the inherent variations in animal species, production systems, and uneven sample collection across regions, residual confounding cannot be fully excluded. In particular, unmeasured factors such as antimicrobial usage, farm infrastructure, educational levels, and access to healthcare resources may have influenced the observed associations. Second, the limited sampling coverage and the absence of long-term, continuous surveillance restrict the generalizability of the findings and preclude the assessment of temporal trends. Third, clonal relatedness among isolates from the same farm was not assessed, and clonal isolates were not excluded; consequently, the effective sample size at the farm level may be overestimated. Finally, regional temperature data were derived primarily from regional meteorological records and may not capture farm-level microclimatic conditions with sufficient resolution.

## Data Availability

The original contributions presented in this study are included in the article/Supplementary Material. Further inquiries can be directed to the corresponding author.

## Supplementary Materials

The following supporting information can be downloaded at: https://www.mdpi.com/article/10.3390/ani16142113/s1, Figure S1. Overall temperature comparison of five regions. A. Comparison of annual average temperatures across five regions. B. Comparison of monthly average temperatures across five regions; Table S1. Antimicrobial susceptibility test interpretive criteria; Table S2. Antibiotic resistance genes primers used in this study; Table S3. The raw data for ARG detection and antimicrobial susceptibility testing of *bla*_NDM_-positive *E. coli* isolates. References [32,33,34,35,36,37,38,39,40,41,42] apply to the contents of the Supplementary Materials.
