# Supplementary material for: Temperature-Associated Prevalence and Multidrug Resistance of blaNDM-Positive E. coli in Livestock Farms in Xinjiang, China
Source: Animals (Basel). 2026 Jul 8;16(14):2113. doi: 10.3390/ani16142113 (PMC13403960; doi:10.3390/ani16142113)
Supplement: Supplementary file 1 [file animals-16-02113-s001.zip › Supplementary Figure S1.pdf]

Supplementary Figure. S1

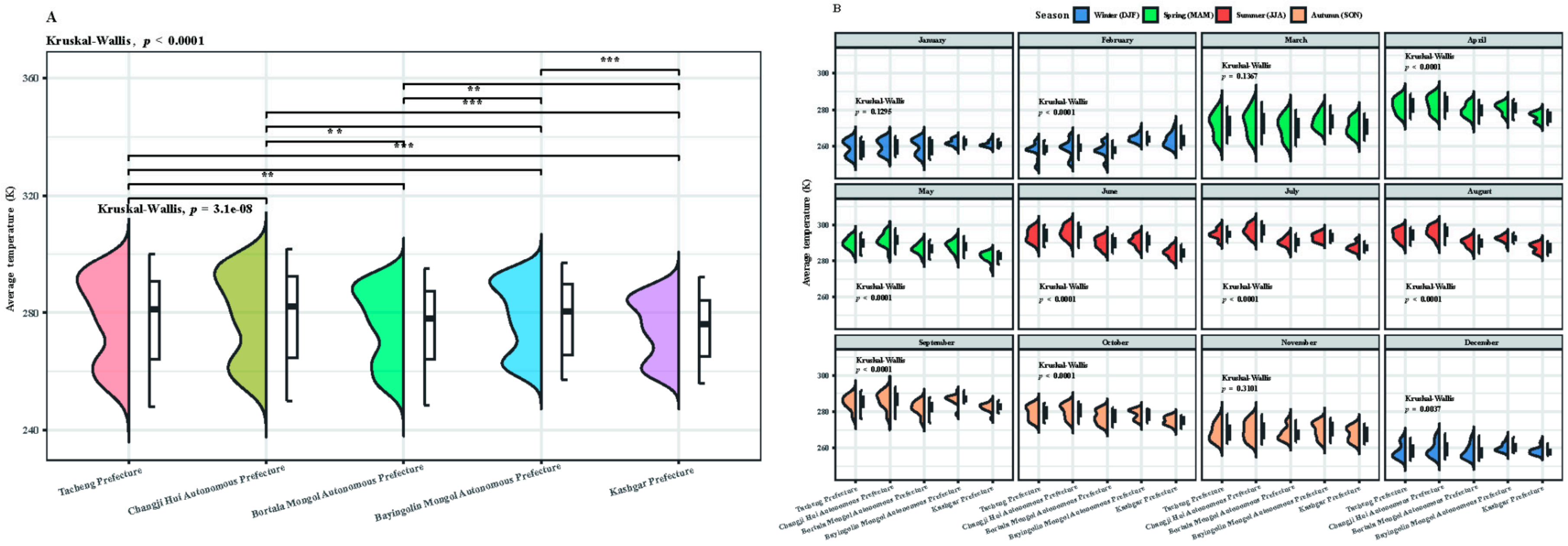

Supplementary Figure. S1 Overall Temperature Comparison of Five Regions. (**A**). Comparison of Annual Average Temperatures Across Five Regions. (**B**). Comparison of Monthly Average Temperatures Across Five Regions.
