# Supplementary material for: Temperature-Associated Prevalence and Multidrug Resistance of blaNDM-Positive E. coli in Livestock Farms in Xinjiang, China
Source: Animals (Basel). 2026 Jul 8;16(14):2113. doi: 10.3390/ani16142113 (PMC13403960; doi:10.3390/ani16142113)
Supplement: Supplementary file 1 [file animals-16-02113-s001.zip › Supplementary Table S1.pdf]

Supplementary Table S1

Antimicrobial Susceptibility Test
Interpretive Criteria

antibiotic agent	MIC breakpoint (μg/mL)			Breakpoint Source
	S	I	R	
imipenem (IPM)	≤1	2	≥4	CLSI （M100-S29）
ampicillin (AMP)	≤8	16	≥32	
ceftiofur (CFF)	≤2	4	≥8	
polymyxin (PE)	≤2	-	≥4	
amikacin (AMK)	≤16	32	≥64	
tetracycline (TET)	≤4	8	≥16	
gentamicin (GEN)	≤4	8	≥16	
tigecycline (TIG)	≤1	-	＞2	EUCAST (V4.0)
enrofloxacin (ENR)	≤0.25	0.5-1	≥2	CLSI （VET01-A4）
florfenicol (FFC)	≤4	8	≥16	
