# Supplementary material for: Temperature-Associated Prevalence and Multidrug Resistance of blaNDM-Positive E. coli in Livestock Farms in Xinjiang, China
Source: Animals (Basel). 2026 Jul 8;16(14):2113. doi: 10.3390/ani16142113 (PMC13403960; doi:10.3390/ani16142113)
Supplement: Supplementary file 1 [file animals-16-02113-s001.zip › Supplementary Table S2 .pdf]

Supplementary Table S2 Antibiotic resistance genes primers used in this study

Gene	Primer sequences (5' - 3')	Product (bp)	Annealing temperature (℃)	References
oqxA	F: CTCGGCGCGATGATGCT<br>R: CCACTCTTCACGGGAGACGA	392	57	[32]
oqxB	F: TTCTCCCCCGGCGGGAAGTAC<br>R: CTCGGCCATTTTGGCGCGTA	513	64	
qnrS	F: ACGACATTCGTCAACTGCAA<br>R: TAAATTGGCACCCTGTAGGC	619	55	[33]
aac(6')-Ib	F: TTGCGATGCTCTATGAGTGGCTA<br>R: CTCGAATGCCTGGCGTGTTT	482	54	[34]
ant(3")-Ia	F: ATCTGGCTATCTTGCTGACA<br>R: TATGACGGGCTGATACTGG	284	54	
tet(A)	F: CATTAATAGGCGCATCGCTG<br>R: TGAAGGTCATCGATAGCAGG	930	53	[35]
tet(M)	F: GGTGAACATCATAGACACCGC<br>R: CTTGTTCGAGTTCCAATGC	401	55	[36]
floR	F: TGAACACGACGCCCGCTAT<br>R: GGACCGCTCCGCAAACAA	750	62	[35]
bla$_{TEM}$	F: ATGAGTATTCAACATTTCCGT<br>R: TTACCAATGCTTAATCAGTGA	861	55	[37]
bla$_{CTX-M}$	F: TTTGCGATGTGCAGTACCAGTAA<br>R: CGATATCGTTGGTGGTGCCATA	544	56	[35]
bla$_{NDM}$	F: GGTTTGGCGATCTGGTTTTC<br>R: CGGAATGGCTCATCACGATC	621	52	[38]
bla$_{SHV}$	F: GGTTATGCGTTATATTCGCC<br>R: TTAGCGTTGCCAGTGCTC	867	55	[39]
mcr-1	F: AGTCCGTTTGTTCTTGTGGC<br>R: AGATCCTTGGTCTCGGCTTG	320	55	[40]
mcr-8	F: TCAACAATTCTACAAAGCGTG<br>R: AATGCTGCGCGAATGAAG	856	53	[34]
sul1	F: CTTCGATGAGAGCCGGCGGC<br>R: GCAAGGCGGAAACCCGCGCC	433	70	[41]
sul2	F: GCGCTCAAGGCAGATGGCATT<br>R: GCGTTTGATACCGGCACCCGT	293	69	
sul3	F: CGTAAATATAACCACCGAT<br>R: CCAAGCCTGAATAAATCTCA	326	55	[42]
